# Analysis of Software Read Cross-Contamination in DNBSEQ Data

**DOI:** 10.3390/biology14060670

**Published:** 2025-06-09

**Authors:** Dmitry N. Konanov, Vera Y. Tereshchuk, Ignat V. Sonets, Elena V. Korneenko, Aleksandra V. Lukina-Gronskaya, Anna S. Speranskaya, Elena N. Ilina

**Affiliations:** 1 Research Institute for System Biology and Medicine, Moscow 117246, Russia; ignatsonets@gmail.com (I.V.S.); lenakorneenko0@gmail.com (E.V.K.); lukina.al98@gmail.com (A.V.L.-G.); hanna.s.939@gmail.com (A.S.S.); ilinaen@gmail.com (E.N.I.); 2 Phystech School of Biological and Medical Physics of MIPT, Moscow Institute of Physics and Technology, Dolgoprudny 141701, Russia; tereshchuk.viu@phystech.edu; 3 Institute of Gene Biology, Moscow 119334, Russia; 4 Vavilov Institute of General Genetics, Russian Academy of Sciences, Moscow 117971, Russia

**Keywords:** DNBSEQ, sequencing artifacts, data filtering, read duplicates

## Abstract

DNA nanoball sequencing (DNBSEQ) is one of the most rapidly developing sequencing technologies and is widely applied in genomic and transcriptomic investigations. Recently, a new PE300 sequencing option primarily recommended for amplicon analysis was released for DNBSEQ-G99 and G400 devices. Given their unprecedentedly high data yield per flow cell, the new PE300 kits could be a great choice for various sequencing tasks, but we found that combining different types of DNA libraries in a single run could lead to undesired artifacts in the data. In this study, we investigate the occasional read cross-contamination that we first observed in our DNBSEQ PE300 run. The phenomenon, which we refer to as “software contamination”, is not actual contamination but primarily manifests as improper forward/reverse read pairing, improper demultiplexing, or as “digital chimeric” reads. Although rare, these artifacts were found in all runs we have analyzed, including several MGI demo datasets (both PE100 and PE150). In this study, we demonstrate that these artifacts arise primarily from the incorrect resolution of sequencing signals produced by neighboring DNA nanoballs, leading to mixing out forward and reverse reads or improper demultiplexing. The artifacts occur most frequently with read pairs where the length of insert sequence is shorter than the read length. Based on a few external NA12878 human exome sequencing data, we conclude that the total improper pairing rate in DNBSEQ data is comparable to Illumina ones. Overall, the problem only affects the analysis results when simultaneously sequenced libraries have markedly different insert size distribution or flow cell loading. Additionally, we demonstrate here that raw DNBSEQ data might contain ~2% optical duplicates, resulting from the same effect of close neighboring of DNB-sites in the flow cell.

## Simple Summary

In this study, we describe in detail certain sequencing artifacts, specific to the MGI DNBSEQ sequencing platform, such as improperly demultiplexed reads and optical duplicates. We provide some recommendations and demonstrate the need for additional data filtering procedures. We hope, that our findings will be helpful for MGI users, and may serve the basis for further development of new DNBSEQ-specific tools.

## 1. Introduction

DNA nanoball sequencing (DNBSEQ) offered by MGI Tech. Co. is a rapidly evolving sequencing platform, now regarded as the primarily alternative to Illumina [1,2,3]. Like Illumina, MGI provides both single-end (SE) and paired-end (PE) sequencing options, with a variety of read lengths from 50 to 400 bases for the SE option, and from 100 to 300 bases for the PE option. In recent years, DNBSEQ technology has proven effective for transcriptomics [4,5], mitochondrial DNA sequencing [6], and genome-wide association studies [7,8].

The PE300 option, released in 2023 for DNBSEQ-G400 and DNBSEQ-G99 devices, is mainly recommended for the sequencing of long amplicon genomic libraries (e.g., 16S rRNA/18S rRNA/ITS), but is also useful in whole-genome sequencing (WGS) since longer fragments might simplify the assembly graph resolution, especially the resolution of complex repeated structures [9]. Finally, the usage of PE300 fragments may enhance the read mapping specificity, which could be useful in RNA-seq or variant calling.

Nevertheless, in DNBSEQ, the library preparation and sequencing schemes that are distinct from Illumina might introduce new types of sequencing errors and artifacts in sequencing signal post-processing, which could not be detected using methods designed for Illumina data. However, due to transparent sequencing logic and read naming, certain platform-specific artifacts could be caught from the data directly. Thus, in cases when the insert is short enough, DNBSEQ reads may stochastically capture not only the insert sequence but also technical MGI sequences, i.e., adapter and barcode (as well as Illumina reads [10]), which allows us to catch chimeric reads or demultiplexing failures. Since the new PE300 kit provides reads much longer than previous paired-end options, PE300 data are expected to allow us to catch significantly more such artifact reads.

Read cross-contamination between samples sequenced in a single flow cell is one of these undesired artifacts, which could complicate further data analysis. Fortunately, in DNBSEQ, based on the read structure, we could explicitly differentiate physical sample cross-contamination from device-based contamination. Physical cross-contamination between samples is a common occurrence [11] and might arise at any library preparation stage and appear as read pairs that are unambiguously mapped to the object from another sample but carry the target barcode sequence.

In this study, we focused on the second case of contamination arising after sample preparation at the sequencing, basecalling, or demultiplexing stages. We found that in raw DNBSEQ data, a number of read pairs are not correctly paired or carry barcode sequences different from the barcode assigned during demultiplexing. Here, we consider in detail this phenomenon that we first faced in our PE300 run and thereafter observed in other DNBSEQ datasets, including MGI demo data.

## 2. Methods

### 2.1. Sequencing Information

In total, there were five library types in the described PE300 run: whole-genome sequencing (WGS) of metagenomes (8 barcodes), WGS of individual bacteria (4 barcodes), RNA metagenomes from bats (20 barcodes), 16S rRNA metagenomic data (4 barcodes), and WGS of artificial plasmids (3 barcodes).

RNA was extracted from twenty bat fecal samples using the QIAamp viral RNA mini kit (Qiagen, Hilden, Germany). The concentration of total RNA was measured using the Qubit RNA HS Assay (Thermo Fisher Scientific, Waltham, MA, USA). The first strand of cDNA was obtained using the NEBNext Ultra II RNA First Strand Synthesis Module (NEB, Ipswich, MA, USA), and the second strand of cDNA was obtained using the NEBNext Ultra II Non-Directional RNA Second Strand Synthesis Module (NEB, Ipswich, MA, USA). The library preparation of double stranded cDNA was performed using the MGIEasy FS DNA Library Prep Kit (MGI, Shenzhen, China).

In addition to RNA libraries, there were three shotgun-type libraries in the PE300 run: the WGS of metagenomes (8 barcodes), the WGS of individual bacteria, and the WGS of artificial plasmids (3 barcodes). The concentration was estimated with Qubit 4.0. and the Qubit dsDNA Quantification Assay Kit (Thermo Fisher Scientific, Waltham, MA, USA). Library preparation was performed using the MGIEasy FS DNA Library Prep Kit (MGI, Shenzhen, China) according to the manufacturer’s protocol.

The fifth library type was 16S rRNA V3-V4 amplicons, which were indexed using a custom scheme including both MGI and Illumina barcodes. DNA was extracted from fecal samples of human and rhesus macaques, and the amplification of V3-V4 16S rRNA regions was conducted using universal Illumina V3-V4 primers (5′ -TCGTCGGCAGCGTCCCTACGGGNGGCWGCAG-3′ and 5′ -GTCTCGTGGGCTCGGGACTACHVGGGTATCTAATCC-3′). To avoid contamination between human and macaque samples, all procedures were performed in different pools.

Circularization was performed individually for six pools described above using the MGIEasy Circularization Kit (MGI, Shenzhen, China) according to the manufacturer’s protocol. Enzymatic digestion products were pooled and were taken to DNB preparation. The obtained DNBs were sequenced on a DNBSEQ-G400 sequencer using the DNBSEQ-G400RS High-throughput Rapid Sequencing Kit (FCS PE300) (MGI, Shenzhen, China) and the DNBSEQG400RS Rapid Sequencing Flow Cell (MGI, Shenzhen, China).

16S rRNA metagenomic libraries (93–96 barcodes) were excluded from the analysis because of a non-standard library indexing scheme.

### 2.2. External Data Acquisition and Barcode Detection

Six random NA12878 exome demo datasets provided by MGI were downloaded from the CNGB database (read archive numbers CNR0077641, CNR0104869, CNR0117180, CNR0138723, and CNR0640481; data obtained from DNBSEQ-G400 and DNBSEQ-T7 devices, with PE100 and PE150 sequencing options). Four random Illumina read archives with NA12878 exome sequencing data were downloaded from the SRA database (read archive numbers SRR088693, SRR11910521, SRR1611178, and SRR2106341). The hg38 genome was used as the human genome reference.

### 2.3. Misdemultiplexing Rate Estimation

In the MGI sequencing technology, if the insert length is less than the read length, the FASTQ record might contain a partial or full sequence of MGI adapters and the barcode sequence. For barcode testing, we considered only reads that contain the full 10-base barcode sequence neighbored by at least one adapter sequence. Despite the recommended insert size in MGI sequencing being higher than the sum length of forward and reverse reads, there are usually a number of reads satisfying this condition. Taking into account the structure of DNA nanoballs, we used the following command to extract the counts of different 10-base sequences representing an actual barcode sequence in the read:

**grep ‘AS’ data 1.fastq | awk -F ‘AS’ ‘print substr($2, 0, 10)’ | sort | uniq -c | sort -n**

Here, AS is a partial adapter sequence located to the left of the barcode. AS is CCAAGCGGTCTTAGGAAGACAA for the forward reads, CGTTCTGTGAGCCAAGGAGTTG for the reverse reads.

**Figure 1:**
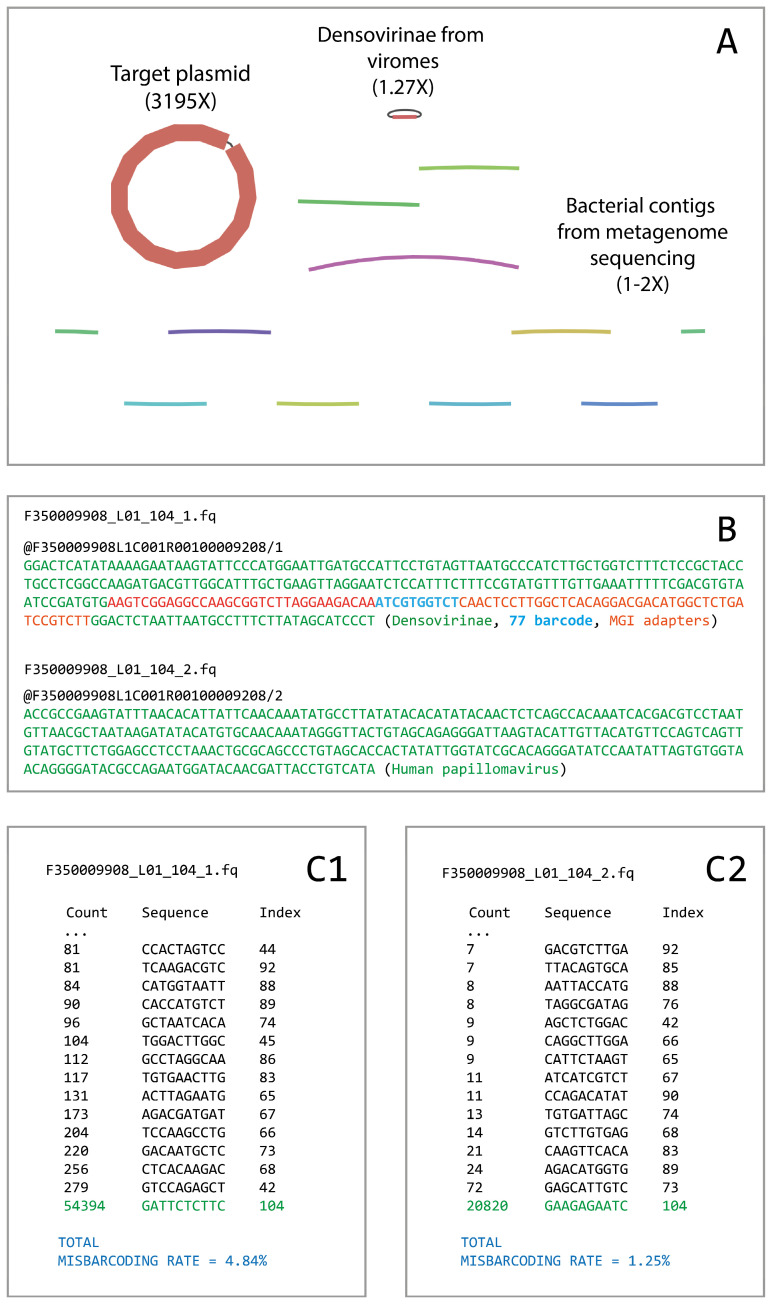
An example of improper read pairing in barcode 104. (**A**) The assembly graph obtained on the reads demultiplexed as barcode 104. The target plasmid with included human papillomavirus has been assembled correctly, but there were a number of contaminating contigs from the objects sequenced under other barcodes. (**B**) An example of a read pair demultiplexed as barcode 104, where the forward read contains a non-target object and a non-target barcode 77 sequence. Here, the reverse read seems to be correctly demultiplexed. (**C1**) The counts of the forward reads in barcode 104 that contained exact barcode sequences different from the target. So, 279 forward reads demultiplexed as barcode 104 actually carried barcode 42. In total, 4.84% of the reads where the barcode sequence could be observed had incorrect barcodes. (**C2**) The same analysis was performed on the reverse reads. The percentage of improperly demultiplexed reverse reads was 1.25%.

Next, we considered only the sequences that are completely identical to one of the barcode sequences used in the considered run. The rate of misdemultiplexing was computed as the percentage of barcode sequences different from the target barcode. Both the bash command and the Jupyterlab notebook for the misdemultiplexing rate estimation are available on Github (github.com/DNKonanov/MGI_manuscript_code (accessed on 1 February 2025)), an example of the processing result is shown in Figure 1(C1,C2).

**Figure 2:**
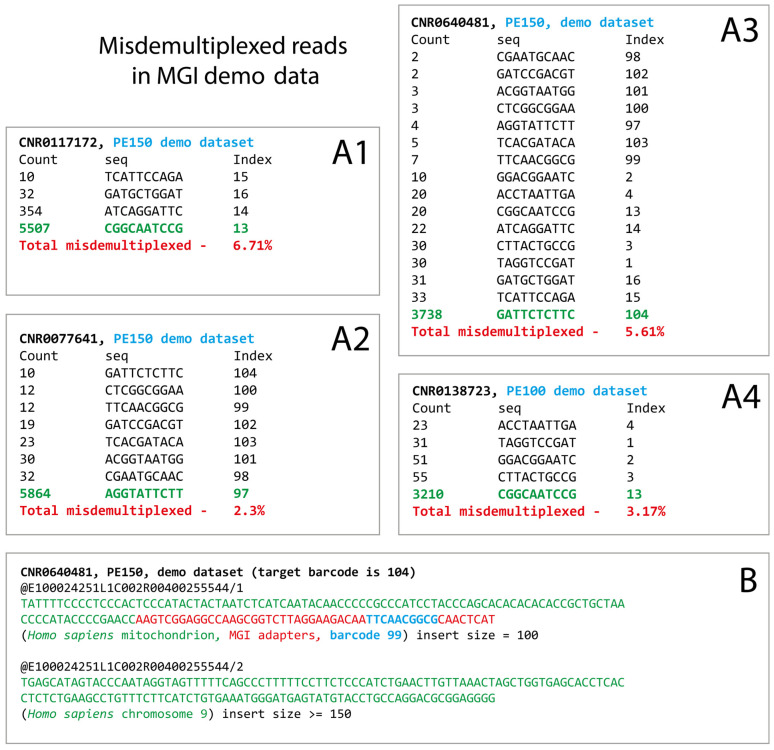
(**A1**–**A4**) Improper read demultiplexing in external demo datasets provided by MGI. Four demo read archives representing the whole-genome sequencing of the NA12878 reference sample (both PE150 and PE100) were downloaded from CNGB for this analysis. It should be noted here that only reads with very short inserts were considered so that they included the MGI technical sequence. (**B**) An example of improper pairing, identical to that observed in our PE300 run.

Since the barcode sequences used in the MGI demo datasets were not stated by the provider, we manually detected them from the data using the same ‘grep’ command. For each external demo dataset, there was only one very frequent 10-mer in the barcode position (the most represented sequence in Figure 2(A1–A4)), so we considered it as the actual used barcode sequence. The validity of this approach can be demonstrated in Figure 1(C1), where the most frequent 10-mer represents barcode 104, which was actually used for this sample. Similarly, for all samples sequenced in the PE300 run, the most frequent 10-mers strictly corresponded to the barcode sequences used (the output of the ‘grep’ for each sample is available in the Github repository https://github.com/DNKonanov/MGI_manuscript_code/tree/main/data/barcode_stats (accessed on 1 February 2025), further confirming the feasibility of this approach.

### 2.4. Mapping Tests

#### 2.4.1. The Association Between Short Insert and Improper Pairing

The raw reads demultiplexed as barcode 46 were divided into two groups. The first group contained only the read pairs where the forward read contained a partial adapter sequence. The second group included the total read set. The reads were mapped to the reference genome using bwa mem [12] (version 0.7.17-r1188, with default parameters). The unmapped reads were selected using sam flag = 4. The same procedure was conducted with raw reads from barcode 104, which were mapped to the resulting plasmid assembly.

#### 2.4.2. The Search for Long Improperly Demultiplexed Reads

The total set of unmapped reads was aligned against the nt database (11 April 2022). Reads with an alignment length greater than 299 were selected. The selected reads were aligned against their mates to divide them into improperly paired and improperly demultiplexed pairs. For the improperly demultiplexed but properly paired reads, the estimated insert sizes were computed using Flash v1.2.11 (with default parameters) [13].

### 2.5. The Analysis of Read Duplicates

The «all-to-all» analysis of duplicated reads was performed in the following way:

1. Based on CxxRxx flag in read identifiers, we divided the total non-demultiplexed FASTQ file into individual camera fields of view (FOVs). Fifty random FOVs were selected for the analysis.
2. For each FOV, we performed the “all-to-all” read mapping using blastn (version 2.9.0+, with default parameters). In preliminary analysis, we mapped the total read set in the FOV to itself, which was rather time-consuming. That allowed us to initially infer the most frequent differences in read IDs. Next, for computational efficiency, each FOV was additionally divided to smaller frames with a size of 1000 reads. Frames were selected in a sliding window manner with window = 500 and mapped to themselves only inside the frame.
3. We considered as duplicates the blast alignments with identity ≥ 95% and an alignment length ≥ read length. The absolute differences in read ID were collected from all detected duplicate pairs to plot the histogram. Zero ID differences were omitted.

The “fast” approach used the assumption that all optical duplicates should belong to one FOV and have identifiers different by 1, 63, 85, 195, or 217 (±2). Pairwise alignment was performed only between reads that had such identifier differences. The number of reads which had duplicates divided by the total number of reads was considered as the rate of optical duplicates.

The Chi-squared statistic was used to estimate the significance of the difference. The effect size was estimated using odds ratios.

## 3. Results

### 3.1. DNBSEQ Forward and Reverse Reads Can Be Improperly Paired or Demultiplexed

We first detected the described phenomena when analyzing our DNBSEQ-400 PE300 run, which included five different types of libraries with different loading to the flow cell (the run details are available in Supplementary Materials S1). During bioinformatic analysis, we observed a small contamination between barcodes which was especially noticeable in the barcodes with lower library loading. In some cases, the contamination could be observed even in the resulting assemblies (Figure 1A).

The analysis of contaminated read pairs showed that, in most cases, these pairs were not properly paired, i.e., the forward and reverse reads undoubtedly carried different inserts (an example is presented in Figure 1B). Moreover, in cases when the insert size was less than 230–240 bases, we observed barcode sequences that were different from the barcode assigned by the basecaller.

Under the assumption that we can partially detect artifact reads using incorrect barcode sequences, we checked all the barcodes presented in the described PE300 run independently. To do that, we selected all the forward reads that included an exact sequence of the partial left MGI adapter CCAAGCGGTCTTAGGAAGACAA, and extracted the following ten bases in the read that should represent barcode sequences. Figure 1(C1,C2) illustrates the analysis of incorrect barcode sequences in the reads demultiplexed as barcode 104. In Figure 1(C1), the list of different 10 bp sequences found on the barcode position in the forward reads is shown. Expectedly, the most frequent variant was the target barcode 104, but there were a number of reads that contained other barcode sequences different from the target. The total percentage of misdemultiplexed forward reads in barcode 104 was estimated to be 4.84%. The mean rate of forward read misdemultiplexing among all barcodes in the total PE300 run was estimated to be 2.54% (std = 1.79).

We performed the same testing for the reverse reads. In general, the percentage of the reverse reads that contained an exact sequence of a non-target barcode was roughly 5-fold lower than for the forward reads. An example for barcode 104 is presented in Figure 1(C2). The total percentage of misdemultiplexed reverse reads in that barcode was estimated to be 1.25%. The mean misdemultiplexing rate for the reverse reads among all used barcodes was 0.56% (std = 0.69). The list of misdemultiplexing rate estimations for each barcode in the PE300 run is available in Supplementary Materials S2.

### 3.2. Improperly Demultiplexed Reads Could Be Found in DNBSEQ Demo Datasets

To extend the analysis, we checked the misdemultiplexing rates in a few demo datasets provided by MGI (accession numbers CNR0077641, CNR0117180, CNR0138723, and CNR0640481; data obtained from DNBSEQ-G400 and DNBSEQ-T7 devices, with PE100 and PE150 sequencing options). For these runs, the barcode sequences were not stated by the data provider and were manually inferred from the data as the most popular barcode sequences located after the MGI adapter. The analysis was conducted in the same manner as in the previous section.

Among reads where technical MGI sequences were found, the estimated rate of misdemultiplexed reads was from 2.3% in CNR0077641 to 6.71% in CNR0117172 (Figure 2(A1–A4)). The observed contaminating barcodes mainly corresponded to the recommended indexing schemes (indices subsets 1–4, 13–16, and 97–104), so their occurrence does not appear to be occasional. Also, we manually found a few examples of improperly paired read pairs identical to those observed in our PE300 dataset (Figure 2B). Therefore, the described artifacts could be found not only in our PE300 results but also in other DNBSEQ data.

### 3.3. Improper Read Pairing Is Mainly Associated with Shorter Inserts

In DNBSEQ, the recommended library length should be at least twice as long as the length of an individual read. In the PE300 run described in this study, the mean library lengths were 460–600 bp depending on the library type, which is less than recommended. Moreover, in the previous section, we tested only read pairs that carried even shorter inserts so that they contained the barcode sequence. Such reads were rather rare in both our and external datasets, and we understood that our estimations could be strongly biased if the reads with shorter inserts are more prone to improper pairing.

We used three different schemes to check the effect of short inserts on the chance of improper pairing or demultiplexing. Firstly, we considered the virome sequencing data from the PE300 run indexed by barcodes 73–92. One of the major assembled organisms in the virome data was the Densovirinae family virus (which will be referred to simply as Densovirinae in the following text). Since it was observed as one of the main contaminants in non-viral barcodes, we decided to consider estimated insert sizes in the read pairs mapped to the Densovirinae (1) in the target virome barcodes and (2) in non-target barcodes.

**Figure 3:**
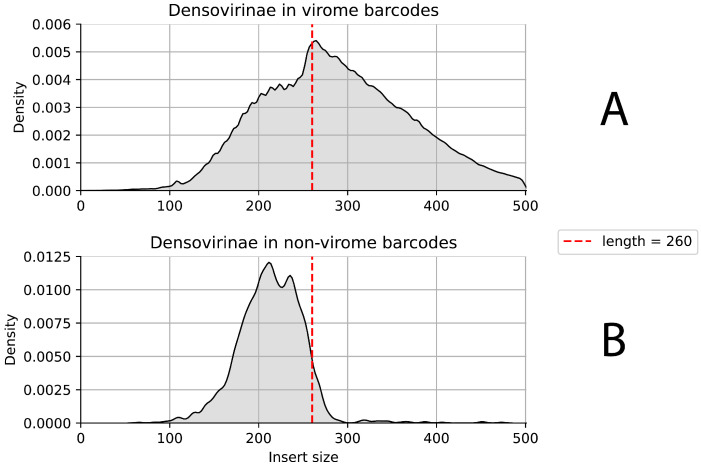
The distribution of estimated insert sizes in read pairs mapped on Densovirinae. (**A**) The insert size distribution in a target barcode 77 (virome). A visible decrease in the number of inserts shorter than 260 can be observed. (**B**) The insert size distribution in a non-target barcode 65 (whole-genome sequencing (WGS) metagenome). Most read pairs that mapped on contaminating Densovirinae had insert size lower than 260.

Indeed, the median insert size in correctly demultiplexed Densovirinae read pairs was significantly longer than the ones in the contaminating read pairs, with a critical value around 260 bp. The inserts with length less than 260 bp are visibly rarer than expected in the target barcodes (Figure 3A) and are mostly presented as contaminating (Figure 3B). The same effect was observed in a few other considered virome barcodes where Densovirinae had high coverage depth (Supplementary Materials S3).

Additionally, we directly tested how the presence of the adapter sequence in forward or reverse read affects the chance of successful mapping. For 46 and 104 barcodes, where the object of sequencing was single (i.e., individual genomic DNA) and known in advance, we divided the read pairs based on the presence of the adapter sequence and mapped the reads from each group to (1) the target genome assembly and (2) the Densovirinae genome. Here, the presence of the adapter sequence means that the insert size is shorter than 268 bases (because the left adapter length is 42 bases). Expectedly, the read pairs with shorter inserts were more rarely mapped to the reference genome (Table 1), and more often mapped to the contaminating Densovirinae genome (Table 2). Thus, we can conclude that shorter inserts, in general, are more prone to improper read pairing or demultiplexing. The described testing schemes were not applicable for external demo datasets since they included only one biological object (the human genome), but we suppose that it is correct for them too.

Thus, the global rate of improper reads pairing or demultiplexing should be less than was estimated in the previous sections. Unfortunately, we cannot clearly distinguish device-based contamination from physical contamination arising in the sample preparation stage when the insert is longer than the read length, so the actual rate of misdemultiplexing remains unknown.

### 3.4. DNBSEQ Data Could Contain a Great Number of Optical Duplicates

**Figure 4:**
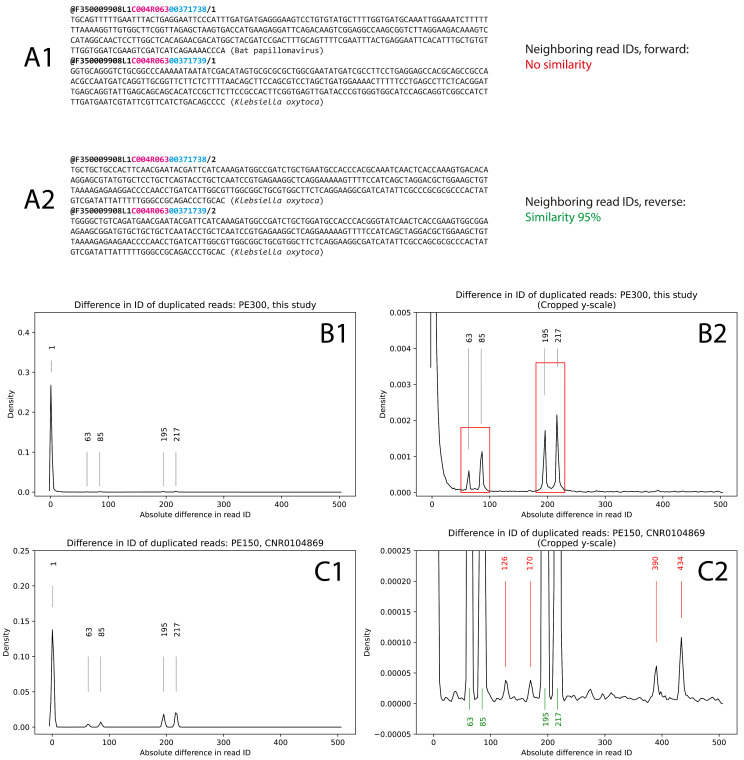
(**A1**,**A2**) An example of incorrect sequencing signal resolution. The neighboring reverse reads (**A2**) are duplicates, while the forward reads (**A1**) with the same IDs do not have similarity. Here, a red substring in the read headers is a field of view (FOV), and blue is a unique read identifier. (**B1**,**B2**) The distribution of the difference between IDs in duplicated reads. The main mode equals 1, so in most cases, duplicated reads have neighboring IDs. Four additional modes might represent farther neighborhoods of DNA nanoballs in the flow cell. (**B1**) has the original y-axis limits, while for (**B2**) the y-axis limits were set so that the four additional modes (indicated by red rectangle) were pronounced. (**C1**,**C2**) The same analysis of an MGI demo dataset (CNR0104869). There, the minor modes were the same but more intense. Four modes marked by red had a doubled difference value compared with those marked by green, probably representing an additional order of neighborhood.

The manual observation of a number of incorrectly demultiplexed or improperly paired reads showed that such events are often associated with the presence of read duplicates or the presence of the reads with neighboring identifiers (an example is presented in Figure 4(A1,A2)). Read identifiers are assigned based on the location of the DNBs in the flow cell, so we supposed that the main source of the improper read pairing is an occasional incorrect resolution of sequencing signals provided by nanoballs located in a close neighborhood.

First, we performed an analysis of duplicated reads regardless of the improper pairing. In order to estimate the general rate of signal mixing, we divided the sequencing data (PE300, total run) by the ‘CxxRxx’ flag in the read identifiers and performed the read duplication analysis in each camera field of view (FOV) independently. Fifty randomly selected FOVs were analyzed. The histogram of absolute differences in duplicated read IDs is shown in Figure 4(B1,B2). Indeed, the read duplicates most frequently had neighboring identifiers (ID difference = 1, major mode). Four reproducible minor modes of 63, 85, 195, and 217 were also observed. Since the positioning of the DNA nanoballs on the flow cell does not follow a strict rectangular grid, these four modes might represent different types of neighborhoods. In the PE300 run, 2.52% reads (280,095 out of 11,131,494 processed reads) had closely located duplicates.

The same procedure was performed on one PE150 demo dataset provided by MGI (accession number CNR0104869). Here, the location of the modes was the same, but the intensity of the additional modes was visibly higher (Figure 4(C1)). Interestingly, each minor mode had its low-intense mate with a doubled ID difference value (Figure 4(C2)). The total percentage of read duplicates with close location was 1.65% (414,661 out of 25,142,640 processed reads).

Since the process of de novo (“all-to-all”) duplicate identification was very time-consuming, the rest of the MGI demo datasets were analyzed in a faster way. We selected random FOVs and performed the pairwise alignment between reads only if they had identifiers different by value from the set [1, 2, 61, 62, 63, 64, 65, 83, 84, 85, 86, 87, 193, 194, 195, 196, 197, 215, 216, 217, 218, 219] which closely represents four observed modes. Compared to the “all-to-all” alignment, the “fast” approach identified about 97% of the duplicated reads in our PE300 run and >94% of the duplicated reads identified in the CNR0104869 demo dataset, so it could be used for the fast estimation of the optical duplicate rate.

The identification of optical duplicates using the “fast” method was performed on four additional demo datasets. The number of reads that had duplicates was divided by the total number of selected reads. The observed rates of optical duplicates in datasets CNR0077641 (PE150), CNR0640481 (PE150), and CNR0138723 (PE100) were 1.632%, 2.096%, and 0.736%, respectively. Dataset CNR0117172 (PE150) had the lowest number of duplicated reads with an estimated rate of 0.208%, which is probably because this dataset had the smallest total number of reads. The results are presented in Table 3.

### 3.5. Improper Pairing or Demultiplexing Is Strongly Associated with Close DNA Nanoball Neighboring

Next, we checked if improper pairing or improper demultiplexing is associated with read duplication. There, we planned to consider four different cases:

1. Improperly paired, short insert.

Read pairs with a short insert; the forward is not overlapped with its mate; incorrect barcode sequence in the forward read.

2. Properly paired, improperly demultiplexed, short insert.

Read pairs with a short insert; the forward is completely overlapped with its mate; incorrect barcode sequence in both forward and reverse reads.

3. Improperly paired, long insert.

Read pairs with a long insert (≥300); the forward is not overlapped with its mate; the forward is not mapped to the reference object; the reverse is mapped to the reference object or another genome different from the forward.

4. Properly paired, improperly demultiplexed, long insert.

Read pairs with a long insert; the forward is completely or partially overlapped with its mate; neither the forward nor the reverse are mapped to the reference object. (Unfortunately, we had to exclude this group from the analysis because we could not distinguish incorrect signal resolution from physical sample cross-contamination.)

For cases “1” and “3”, we selected read pairs with chosen properties and checked the number of reverse reads that had a neighboring duplicate. In total, 23.91% and 23.99% of reads from the first and third groups, respectively, had neighboring duplicates compared to 2.516% in the total read set (Chi-square *p*-value = 10^−308^ and 10^−18^, respectively, with odds ratios of 9.50 and 9.53, respectively). For case “2”, we also selected the read pairs with chosen properties and checked if the reverse read had a close neighbor demultiplexed to the considered barcode, with read ID different by 1–2, 61–65, 83–87, 193–197, and 215–219. In total, 26.09% of the selected read pairs had such a neighbor, compared to 7.51% in the total read set (*p*-value = 10^−8^; odds ratio = 3.47). Thus, we can conclude that the observed improper pairing and improper demultiplexing events are strongly associated with read duplication, which is likely caused by DNA nanoball neighboring.

### 3.6. Incorrect Signal Resolution Might Be a Source of “Digital Chimeras”

**Figure 5:**
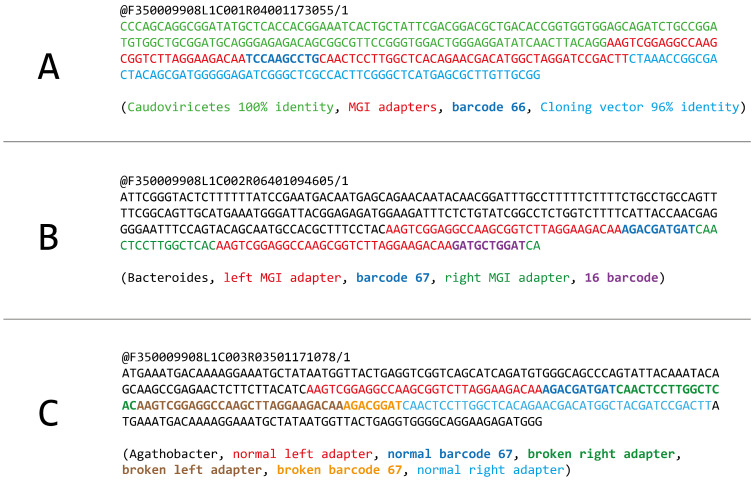
Examples of chimeric and broken forward reads in the DNBSEQ-G400 PE300 run. (**A**) An example of a chimeric read that contains two different inserts. (**B**) An example of a read containing two different barcode sequences. (**C**) An example of a read with a broken technical sequence.

During the analysis of our PE300 run, we also observed a few types of reads with an unexpected chimeric structure. The first type were reads that contain two different inserts mapped to different objects (Figure 5A). The second type were reads that contain two different barcode sequences (Figure 5B). The third type were reads that have a broken technical sequence (Figure 5C). Although the last ones are not literally chimeric, we decided to mention them here. Since the structure of chimeric reads could be very fluid, we did not manage to suggest any formal rules for their automated search, and all the presented examples were found manually.

Generally, these chimeric reads might be caused by either the chimeric structure of DNA nanoballs or incorrect sequencing signal resolution. We think that incorrect signal resolution is more likely the chimera source, since nanoballs with a non-regular structure cannot produce a homogenous sequencing signal, so this is why we call them “digital chimeras”.

It is difficult to accurately estimate the actual percentage of chimeric reads because they do not share structural features that can be easily detected. However, in our opinion, they are extremely rare, and their presence does not affect the results of data analysis. Again, the reads can be identified as chimeric only if they carry inserts shorter than the read length.

In external datasets analyzed in this study (PE100 or PE150 runs), read lengths were not long enough to catch similar events, while PE300 demo datasets with non-amplicon sequencing data were not publicly available in the China National GeneBank (CNGB) at the time of conducting this study, so we cannot declare that these chimeric structures are characteristic of external runs.

### 3.7. The Actual Rate of Improper Read Pairing in MGI Data Is Similar to Illumina but the Percentage of Optical Duplicates Is Higher

A certain number of improperly paired reads and optical duplicates could be found in paired-end raw data obtained using any sequencing technology. Analogically to MGI external data, we downloaded from the NCBI SRA a few human exome datasets (human reference sample NA12878 [14]) generated on different Illumina devices (read archive numbers SRR088693 [15], SRR11910521 [16], SRR1611178 [17], and SRR2106341 [18]) and compared the rate of improper pairing with MGI data using the ‘samtools stats’ command. To ensure the consistency of the analysis, in this section, both DNBSEQ and Illumina data were analyzed using ‘samtools stats’ with default parameters. The resulting percentage of discordantly mapped read pairs in MGI data was from 1.1 to 3.2%. In Illumina data, the observed percentage of improperly paired reads was from 1.0 to 3.4%, so there is no significant difference between the technologies in that context. It should be noted that these percentages may not be consistent with the improper pairing rates obtained above since the ‘samtools stats’ command uses all the read pairs from the data, while, in the previous sections, only reads with short insert sizes were used for the testing.

Contrary to DNBSEQ, demultiplexing algorithms for paired-end Illumina data use both forward and reverse reads, so we cannot expect the same misdemultiplexing events for the forward reads. However, for analysis consistency, we checked the misdemultiplexing rates based on short inserts in a couple of Illumina datasets in the same manner and did not find any cases of improper demultiplexing (an example is available in Supplementary Materials S4).

Additionally, for the downloaded Illumina read archives SRR1611178 and SRR2106341 (where read identifiers had an appropriate format with the positioning: x: y information), we checked the percentage of optical duplicates using Picard MarkDuplicates. The resulting percentages were 0.012% and 0.015%, respectively, which are significantly lower than what was detected in both our and external MGI data.

## 4. Discussion

DNBSEQ PE300 sequencing has great potential as a high-throughput and rapid sequencing technology. However, while preparing this manuscript, we found that very few studies using the MGI PE300 have been published since the PE300 protocol was released [19,20]. To our knowledge, this is the first detailed technical description of an MGI PE300 run, and we hope that our observations will be helpful for MGI users.

The amplicon sequencing, for which PE300 is first recommended, does not require such data output [21] as is generated by DNBSEQ-G99 or DNBSEQ-G400 devices. But users should be aware that combining amplicons with other DNA libraries, especially those with different insert sizes and loading in a single run, may lead to visible artifacts in the data.

In this study, we examined the main scenarios when such artifacts arise and their potential sources. We deliberately focused on our PE300 run because it included multiple different libraries loaded in one flow cell, and the described effects were pronounced most clearly. We found that read pairs with shorter inserts are more prone to improper pairing or demultiplexing. For some reason, in DNA nanoballs with short inserts, the synthesis of the forward read could pass successfully, but the synthesis of the reverse read or the barcode sequence might occasionally fail, and the basecaller merges signals from neighboring nanoballs to one read pair, thus generating improperly paired or chimeric reads. Thus, in data preprocessing, it seems useful to filter all the read pairs where the forward read includes an adapter sequence. In particular, in the PE300 run considered in this study, such filtering (using Cutadapt [22] with a --discard-trimmed option) removed most contaminating Densovirinae reads from other barcodes. Since this procedure does not alter the FASTQ structure, it could be combined with other state-of-the-art techniques to preprocess raw data.

We should note that reads resulting from inserts longer than the read length in very rare cases might also be improperly paired or demultiplexed, but these events could be detected only indirectly by mapping reads to the sequenced objects and only when the sequenced objects belong to different taxa. However, in such instances, distinguishing between contamination from improper signal processing and cross-contamination during library preparation remains challenging.

Chimeric reads also occasionally appear in the DNBSEQ data. These are difficult to detect because their structure can vary from reads containing two different inserts to those with a complex mosaic structure including different forms of complete or partial barcodes and sequencing adapters. We suppose that chimeric reads could have originated either in the DNA nanoball preparation stage (due to errors in rolling circle amplification) or as a result of probably incorrect signal resolution during the sequencing process, each producing distinct chimera topologies. Fortunately, chimeric reads are extremely rare, and, based on our analysis, do not significantly affect any post-processing procedures. As with improper pairing, chimeric reads are only identifiable in cases when the insert is shorter than the read length, so the filtering of reads containing technical sequences allows the removal of most observed chimeric variants from the data.

The close DNA nanoball neighboring is pronounced in another way. Similarly to Illumina’s non-optimal clustering effect [23], read duplicates could be produced for the closely neighboring flow cell sites. In the described cases, since there was no classical PCR process while library preparation, the duplicates most likely have an optical nature, and we are able to explicitly distinguish them due to the transparent naming of the read IDs. The number of duplicated reads likely depends on the total amount of DNA nanoballs per flow cell, as was demonstrated on external datasets (Table 3), and possibly on the insert copy number in them. Most closely located duplicated reads have IDs in output FASTQ files different by 1, but four additional types of neighborhoods were inferred (ID difference = 63, 85, 195, 217). The estimated percentage of optical duplicates in both our data and external demo datasets was around 2%, which is significantly higher than typical Illumina values. However, we understand that the estimations should not be directly compared because different approaches were used to identify the optical duplicates in MGI and Illumina data.

In WGS analysis, deduplication should be used to clean the data, as well as for Illumina datasets [24], but the filtering of amplicon sequencing data, for which PE300 is primarily recommended, is not so clear. Thus, the dada2 tool [25], widely used for the construction of amplicon sequence variants (ASVs), considers a sequence as a potential individual ASV only if there are at least two identical copies of that particular sequence in the data. As a result, optically duplicated reads could lead to redundant ASVs in the results.

Fortunately, when the mean length of the sequenced amplicon is at least twice the read length, the appearance of artifact read pairs is not expected, since the longest insert size for which we observed improper signal assignment was around 450 bp in the PE300 run. However, the number of optical (closely located) duplicates is still rather high even in the libraries with long inserts, which was shown on MGI demo data (accession number CNR0104869). It should be noted that deduplication tools such as the ‘MarkDuplicates’ module from Picard are designed for Illumina reads and are not capable of distinguishing between optical and PCR duplicates in DNBSEQ data, because the structure of the FASTQ headers is different [26]. To our knowledge, this is the first study where the presence of optical duplicates in DNBSEQ data was demonstrated. Thus, in earlier works, they were not identified by Picard, which reported 0% of optical duplicates for DNBSEQ data [27], which highlights the need to develop optical duplicate detection methods compatible with the FASTQ header structure used by MGI.

Illumina, to which DNBSEQ is positioned as an alternative, uses a different approach to the demultiplexing of paired-end reads using forward and reverse reads themselves, while DNBSEQ uses only an additional barcode fragment sequenced using a special barcode primer. Due to that, in Illumina data, we did not observe the improper demultiplexing of forward reads, which we estimated in the same manner as for DNBSEQ data, which demonstrated that such device-based improper demultiplexing is most likely specific for DNBSEQ technology.

Despite all the described issues, the overall percentage of discordant read pairs in DNBSEQ data does not differ from Illumina results when sequenced libraries are homogenous and have been prepared following the MGI recommendations (as shown on MGI demo data and a few external Illumina datasets). Indeed, the effect of software contamination has a stochastic nature, and given that each barcode has the same probability of incorrect signal resolution, the minimal observed percentage of artifact reads is reached when concentrations of barcodes in the run are equal. Therefore, to minimize the effect of artifact reads on data processing, it is necessary to strictly follow the insert size recommendation for the given sequencing kits and not mix different types of DNA libraries in a single flow cell.

## 5. Conclusions

DNBSEQ might occasionally produce improperly paired or improperly demultiplexed read pairs, which could result in the appearance of contaminating read pairs and contaminating contigs in the final assemblies. Generally, only read pairs with inserts shorter than those recommended by MGI are prone to the described artifacts, so it seems useful to remove reads with short inserts from the data using tools such as Cutadapt. On the other hand, due to technical reasons, a formal search for artifactual reads is available only for reads with short inserts, and the detection of improperly paired or demultiplexed reads with long inserts remains challenging.

The authors suppose that the main source of the artifacts is the incorrect resolution of the sequencing signals obtained from neighboring DNA nanoball sites. A related problem is the presence of optical duplicates in DNBSEQ data, which might complicate amplicon sequencing results. Since the existing tools for optical duplicate detection are not compatible with MGI DNBSEQ FASTQ headers, further development of new DNBSEQ-specific methods to filter data is required.

## Figures and Tables

**Table 1:** Percentage of forward reads in CA * and not CA group, not mapped to the target genome.

Alignment	Total Number of Forward Reads	Unmapped % in Total Reads	Number of CA Reads	Unmapped % in CA Reads
46 to *K. oxytoca* genome	816,540	0.611%	244,924	1.37%
104 to the plasmid assembly	373,299	3.98%	71,947	6.02%

* CA (contain adapter)—the group of reads resulting from inserts that are short enough that these reads contain an adapter sequence.

**Table 2:** Percentage of forward reads in CA and not CA group, mapped to the contaminating Densovirinae genome.

Alignment	Total Number of Forward Reads	Mapped % in Total Reads	Number of CA Reads	Mapped % in CA Reads
46 to Densovirinae genome	816,540	0.0481%	244,924	0.106%
104 to Densovirinae genome	373,299	0.0653%	71,947	0.216%

**Table 3:** Optical duplicates in DNBSEQ demo datasets.

Accession Number	Run Type, Amount of Data	Device	Method	Optical Duplicates, % Reads
This study	PE300, 200 Gb	DNBSEQ-G400	“all-to-all”	2.52
			Fast	2.44
CNR0104869	PE150, 106 Gb	DNBSEQ-G400	“all-to-all”	1.65
			Fast	1.55
CNR0077641	PE150, 118 Gb	DNBSEQ-G400	Fast	1.63
CNR0640481	PE150, 48 Gb	DNBSEQ-T7	Fast	2.10
CNR0117172	PE150, 25 Gb	DNBSEQ-G400	Fast	0.208
CNR0138723	PE100, 28 Gb	DNBSEQ-G400	Fast	0.736

## Data Availability

The main scripts used in the study, the *K. oxytoca* raw reads, and its genome are available in the GitHub repository (https://github.com/DNKonanov/MGI_manuscript_code (accessed on 1 February 2025)). The same GitHub repository contains demo data and the main scripts for processing read duplicates. Additionally, the genomes of *Klebsiella oxytoca* 3238 and *Klebsiella grimontii* 2750 and non-demultiplexed reads used in this study are available in the NCBI bioproject with accession number PRJNA1158576. The SRR30664047 read archive intentionally contains non-demultiplexed data so that it can be used for duplicate identification. The external Illumina datasets used in this study are available in the SRA database with accession numbers SRR088693, SRR11910521, SRR1611178, and SRR2106341. The external DNBSEQ datasets used in this study are available in the CNGB database with accession numbers CNR0077641, CNR0104869, CNR0117180, CNR0138723, and CNR0640481.

## Supplementary Materials

The following supporting information can be downloaded at: https://www.mdpi.com/article/10.3390/biology14060670/s1, Supplementary S1: PE300 run details; Supplementary S2: estimation of demultiplexing rates in PE300 data; Supplementary S3: additional examples of the short inserts effect demonstrated in Figure 3; Supplementary S4: search for demultiplexing artifacts in Illumina data.
