# Supplementary material for: Analysis of Software Read Cross-Contamination in DNBSEQ Data
Source: Biology (Basel). 2025 Jun 9;14(6):670. doi: 10.3390/biology14060670 (PMC12189395; doi:10.3390/biology14060670)
Supplement: Supplementary file 1 [file biology-14-00670-s001.zip › biology-3679995-supplementary.pdf]

Supplementary Materials for "The Analysis of Software Cross-Contamination in DNBSEQ Data"

1. PE300 run details

There were five library types in the PE300 run: WGS of metagenomes (8 barcodes), WGS of individual bacteria (4 barcodes), RNA metagenomes from bats (20 barcodes), 16s metagenomic data (4 barcodes), and WGS of artificial plasmids (3 barcodes).

The mean insert sizes were measured in pools. The run loading in % was calculated based on the required amount of data for each barcode.

Barcode	Library type	% in run	mean insert size in pool
42	WGS metagenome	6,30%	507
43	WGS metagenome	6,30%	507
44	WGS metagenome	6,30%	507
45	WGS metagenome	6,30%	507
65	WGS metagenome	6,30%	507
66	WGS metagenome	6,30%	507
67	WGS metagenome	6,30%	507
68	WGS metagenome	6,30%	507
48	WGS individual bacteria	0,33%	465
41	WGS individual bacteria	0,33%	465
46	WGS individual bacteria	0,33%	465
47	WGS individual bacteria	0,33%	465
73	RNA metagenome	2%	461
74	RNA metagenome	2%	461
75	RNA metagenome	2%	461
76	RNA metagenome	2%	461
77	RNA metagenome	2%	461
78	RNA metagenome	2%	461

79	RNA metagenome	2%	461
80	RNA metagenome	2%	461
81	RNA metagenome	2%	461
82	RNA metagenome	2%	461
83	RNA metagenome	2%	461
84	RNA metagenome	2%	461
85	RNA metagenome	2%	461
86	RNA metagenome	2%	461
87	RNA metagenome	2%	461
88	RNA metagenome	2%	461
89	RNA metagenome	2%	461
90	RNA metagenome	2%	461
91	RNA metagenome	2%	461
92	RNA metagenome	2%	461
93	16s metagenome (Rhesus gut)	1,20%	526
94	16s metagenome (Human gut)	1,60%	600
95	16s metagenome (Human gut)	1,60%	600
96	16s metagenome (Human gut)	1,60%	600
104	Plasmid	0,30%	470
121	Plasmid	0,30%	470
122	Plasmid	0,30%	470

2. **The estimated misdemultiplexing rates for each barcode in the PE300 run (only short inserts).**

library type	Index number	Md* rate in forward reads	Md* rate in reverse reads	Number of forward reads with exact barcode sequence	Number of reverse reads with exact barcode sequence
wgs metagenomes	42	1.51%	0.31%	4799393	1575615
	43	0.08%	0.01%	721719	401868
	44	2.15%	0.32%	2049531	764418

	45	3.71%	0.79%	1933276	651929
	65	2.76%	0.49%	2222598	754232
	66	1.92%	0.22%	2078544	702973
	67	1.27%	0.22%	3757871	1428055
	68	0.91%	0.1%	3095884	1200687
bacteria	41	7.04%	2.76%	20282	6298
	46	1.71%	0.49%	192786	60333
	47	3.52%	1.15%	75240	18791
	48	2.78%	0.92%	220146	69937
viromes	73	1.42%	0.23%	1006645	500189
	74	1.21%	0.1%	1274957	605771
	75	2.29%	0.27%	785660	345108
	76	1.61%	0.22%	871336	341681
	77	1.43%	0.21%	998777	412973
	78	1.82%	0.26%	1462596	661921
	79	3.03%	0.71%	434316	164141
	80	8.41%	1.99%	259018	85300
	81	2.48%	0.39%	514137	235614
	82	2.37%	0.27%	512193	225202
	83	2.1%	0.29%	608697	251354
	84	1.73%	0.21%	874144	388697
	85	1.57%	0.2%	1116461	527364
	86	1.81%	0.24%	866633	385674
	87	1.86%	0.27%	742732	365479
	88	2.14%	0.34%	1389366	643877

	89	1.66%	0.19%	776627	335098
	90	1.59%	0.24%	986116	455138
	91	1.43%	0.2%	1165070	517730
	92	1.46%	0.18%	953817	385509
	94	3.52%	0.6%	641937	359088
plasmids	104	4.84%	1.25%	57158	21084
	121	2.75%	0.62%	71863	20521
	122	7.66%	3.08%	40173	16130

* Md rate - misdemultiplexing rate

3. Densovirinae as a target organism and as a contamination.

To check if contaminating read pairs carry shorter inserts, we consider the read pairs mapped on the Densovirinae family virus genome that was the most presented in a few virome barcodes. Using ‘bwa mem’, we independently mapped the reads from virome and non-virome barcodes to the assembled Densovirinae. Next, we extracted only read pairs where both forward and reverse were mapped with MAPQ = 60, and excluded the read pairs with estimated insert size equals 0, or greater than 500 (to filter outliers).

The insert size distribution for the selected read pairs from target (74-79) and non-target (46 and 65) barcodes are shown on the **Supplementary Figure X**.

Indeed, the median insert size in contaminating read pairs is clearly lower than in the correctly demultiplexed read pairs.

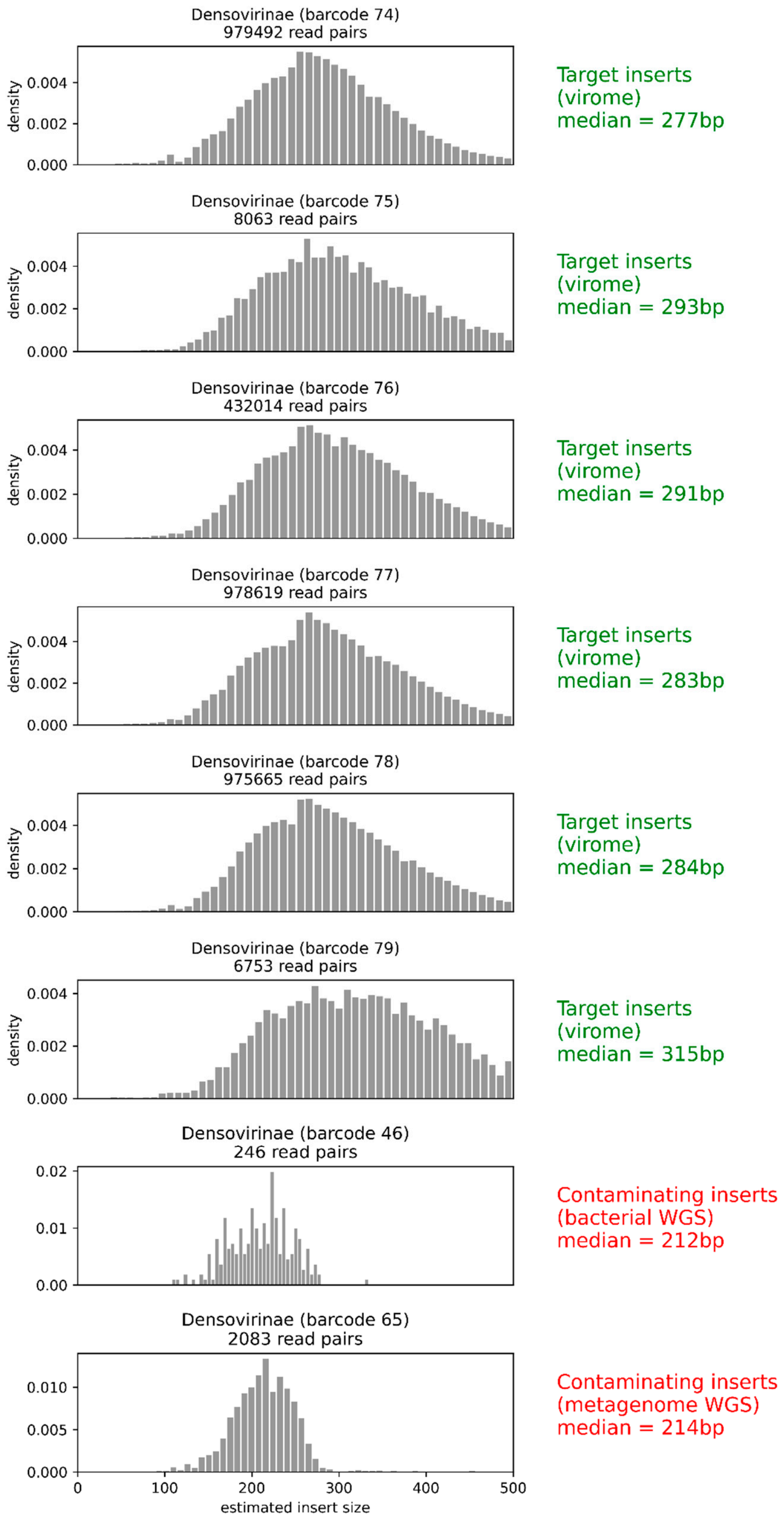

Supplementary Figure S1. The insert size distribution in six virome barcodes (74-79), and two non-virome barcodes (46 and 65). In 74, 76, 77, and 78 barcodes where Densovirinae was a major organism, a visible step on the histogram around 260bp could be observed. The insert sizes in the non-target barcodes are significantly lower.

4. **Testing misdemultiplexing rate in Illumina data.**

Using the same logic we checked the misdemultiplexing rate in Illumina data. The command for extracting presented barcodes was as follows:

```
grep ‘GATCGGAAGAGCACACGTCTGAACTCCAGTCAC’ data.fq | awk -F
‘GATCGGAAGAGCACACGTCTGAACTCCAGTCAC’ ‘{print substr($2, 0, 8)}’
| head -n 10000 | sort | uniq -c | sort -n
```

In all analyzed barcodes, we did not identify exact sequences of barcodes different from the target barcode. The examples of detected sequences in a few barcodes are shown below.

```
106_S5_R1_001.fastq
      ...
      4 ACTCAGAA
      5 ATTCAGGA
      5 CTTCAGAA
      6 ATTCAGAC
      8 ATTCAGCA
     11 ATTCCGAA
     15 ATTCATAA
     29 ATTCAAAA
     35 ATTAAGAA
   9858 ATTCAGAA target barcode, 0.0% misdemultiplexed
-
148_S12_R1_001.fastq
      ...
     10 GAGATGCC
     10 GAGATTTC
     15 GCGATTCC
     17 GAGAATCC
     19 GAGATTAC
     19 GAGCTTCC
     23 AAGATTCC
     31 GAAATTCC
     46 GAGATTCA
   9715 GAGATTCC target barcode, 0.0% misdemultiplexed
-
160_S33_R1_001.fastq
      ...
      4 ATTACGCG
      7 ATTACACG
      9 AATACTCG
     14 ATTACTCC
```

```
     15 CTTACTCG
     16 ATTCCTCG
     18 ATTACTCT
     21 ATTACTCA
     23 ATTACCCG
     24 ATTACTAG
     40 ATTAATCG
   9788 ATTACTCG target barcode, 0.0% misdemultiplexed
-
161_S3_R1_001.fastq
      ...
     14 CGCTCATA
     16 CCCTCATT
     16 CGATCATT
     17 CGCTCAAT
     18 CGCGCATT
     20 CGCTAATT
     23 AGCTCATT
     47 CGCCCATT
     64 CGCTCCTT
   9669 CGCTCATT target barcode, 0.0% misdemultiplexed
```
